# Short-Term Cast Immobilization of a Unilateral Lower Extremity and Physical Inactivity Induce Postural Instability during Standing in Healthy Young Men

**DOI:** 10.3390/healthcare11182525

**Published:** 2023-09-13

**Authors:** Takuro Ikeda, Shinichiro Oka, Junya Tokuhiro, Akari Suzuki, Kensuke Matsuda

**Affiliations:** 1 Department of Physical Therapy, Faculty of Medical Sciences, Fukuoka International University of Health and Welfare, Fukuoka 814-0001, Japan; 2 Department of Physical Therapy, Faculty of Rehabilitation, Reiwa Health Sciences University, Fukuoka 811-0213, Japan; s.oka@kyoju.ac.jp; 3 Department of Rehabilitation, Gotanda Hospital, Oita 877-0037, Japan; 1462056@g.iuhw.ac.jp; 4 Department of Physical Therapy, Faculty of Health Sciences at Fukuoka, International University of Health and Welfare, Fukuoka 831-8501, Japan; akari_s@iuhw.ac.jp (A.S.); k.matsuda@iuhw.ac.jp (K.M.)

**Keywords:** center of pressure, cast immobilization, movement restriction, physical inactivity

## Abstract

Previous studies have reported an increased postural sway after short-term unilateral lower limb movement restriction, even in healthy subjects. However, the associations of motion limitation have not been fully established. The question of whether short-term lower limb physical inactivity and movement restriction affect postural control in the upright position remains. One lower limb of each participant was fixed with a soft bandage and medical splint for 10 h while the participant sat on a manual wheelchair. The participants were instructed to stand still for 60 s under eyes-open (EO) and eyes-closed (EC) conditions. Using a single force plate signal, we measured the center of pressure (COP) signal in the horizontal plane and calculated the total, anterior–posterior (A–P), and medial–lateral (M–L) path lengths, sway area, and mean COP displacements in A–P and M–L directions. The COP sway increased and the COP position during the upright stance shifted from the fixed to the non-fixed side after cast removal, compared to before the cast application, under both EO and EC conditions. These findings indicated that 10 h of unilateral lower limb movement restriction induced postural instability and postural control asymmetry, suggesting the acute adverse effects of cast immobilization.

## 1. Introduction

Postural control is based on the integration of sensory information from the visual, somatosensory, and vestibular senses to maintain equilibrium [1,2]. This afferent sensory information is transmitted to multiple brain regions that monitor limb position and control muscles and joints to minimize body sway [3]. Thus, postural control requires complex interactions between the nervous and musculoskeletal systems. Disorders of any of these systems can increase the risk of falls in older adults and decrease the performance of athletes [4,5]. Therefore, researchers have attempted to understand the physiological and kinematic factors that influence postural balance disorders [1,2,3]. Investigating the factors that influence postural instability is necessary in order to develop more effective system disorder prevention and remediation programs.

In general, musculoskeletal impairments (e.g., ligament injury) and aging (i.e., sarcopenia) impair standing postural stability [6,7]. In addition, a systematic scoping review revealed that balance coordination is affected even after dozens of days of bed rest with the head tilted down [8]. Rest is an intervention frequently used for general recovery and tissue repair in patients with illness or injury. These studies suggest that postural instability may occur after improvements in the condition.

Clinically, in cases of trauma to only one of the lower limbs a plaster cast or splint may be used to partially restrict the movement of that limb to promote tissue repair as a conservative treatment after surgery [9]. Caplan et al. [10] reported that in asymptomatic adult volunteers, one week of unilateral ankle immobilization decreased balance. Elam et al. [11] reported that postural sway in healthy older adults increased after two weeks of unilateral above-knee cast immobilization. Following lower limb surgery, patients may experience postural instability due to secondary dysfunction (i.e., muscle weakness and reduced range of motion) [12]. The reports by Caplan et al. [10] and Elam et al. [11] suggest that movement restriction of the unilateral limb itself is a factor that inhibits postural control. Recently, we observed that in asymptomatic healthy adults, standing postural sway was increased after 10 h of above-knee cast immobilization compared to before wearing the cast, and the center of pressure (COP) was deviated to the non-fixed limb side [13]. An important finding was that these COP changes occurred during the early stages of immobilization. Several neurophysiological studies have reported that approximately 10 h of unilateral limb immobilization may induce synaptic depression in the somatosensory and motor cortex [14]. The COP may change during the upright stance due to modifications of limb representation in the nervous system after limb movement restrictions. Plasticity changes in the nervous system may inhibit the feedback and feed-forward processes necessary for posture control [15]. However, during the 10 h period, the patients were allowed to move freely using crutches but without the immobilized lower limb contacting the floor. Muscle fatigue increases postural asymmetry and decreases balance while standing still [16]. To date, the possibility that COP sway and deviation are influenced by fatigue has not been ruled out, and the association between postural control and motion limitation has not been fully established.

This study aimed to investigate the acute effects of lower limb physical inactivity and movement restriction on static postural control after cast removal in healthy young men to inform future studies investigating the effects of balance after unilateral lower limb conservative treatment following surgery. We assumed that postural sway and COP directional specificity after cast removal would be greater than those before cast application, as in previous studies on crutch-walking participants.

## 2. Materials and Methods

Written informed consent was obtained from all the participants. The local ethics committee of the International University of Health and Welfare approved this study (16-Ifh-041). The study conformed to the principles of the Declaration of Helsinki.

### 2.1. Participants

Sixteen healthy male adult students (age, 21.3 ± 0.5 years; height, 171.3 ± 4.9 cm; weight, 66.8 ± 8.7 kg) at the International University of Health and Welfare were enrolled. The inclusion criteria were: (a) age between 21 and 30 years, (b) no history of participation in movement restriction studies, and (c) no history of strenuous activity and alcohol consumption for 24 h prior to the experiment. The reason for including only male participants was that females have been reported to experience increased postural sway during ovulation, and postural sway and ankle muscle characteristics during ovulation were found to be interrelated [17]. The exclusion criteria were: (a) a history of injuries or diseases that influence balance and foot sensory function, (b) intake of medications that affect the postural control system, and (c) current involvement in physical training.

### 2.2. Movement Restriction and Physical Inactivity

**Figure 1:**
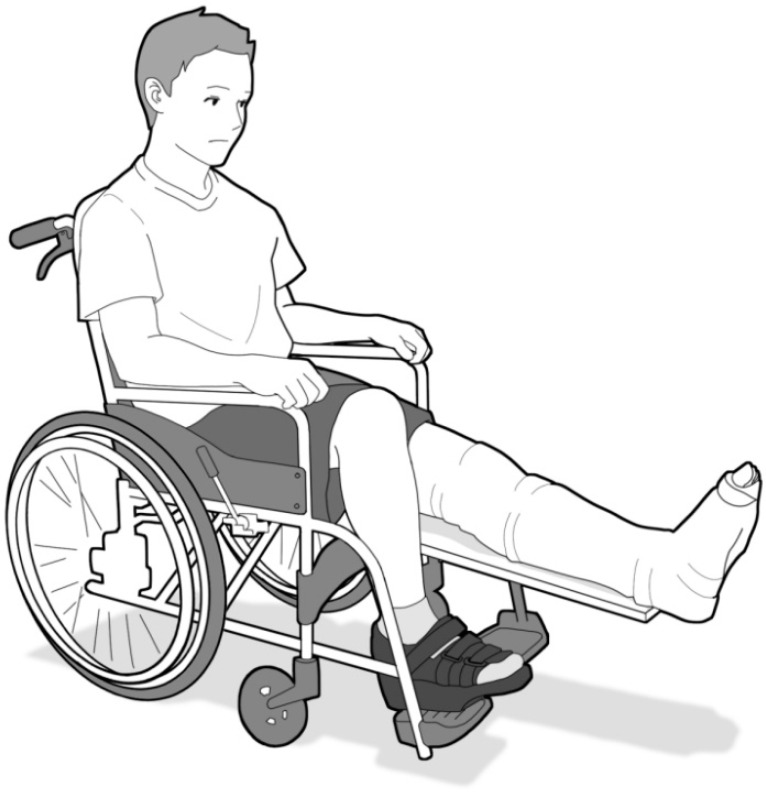
Movement restriction and physical inactivity conditions.

The non-dominant legs of the participants were immobilized from the lower 1/3rd of the thigh to the proximal phalanx using a soft bandage and a medical splint (Soflatsine II; Taketora Corp., Yokohama, Japan). The medical splint is made of galvanized iron wire formed into a ladder shape, covered with polyurethane foam, and laminated with dry sheet fabric. The joint angles were 30° flexion for the knee joint and vertical position for the ankle joint. The question in the survey [18], “Which leg do you kick the ball with?”, was used to determine the non-dominant leg (fixed side). The participants sat on a manual wheelchair (Wish CS-10; Care-Tec Japan Corp., Tokyo, Japan) and were unable to move their immobilized lower limb for 10 h from 09:00 to 19:00. In patients with musculoskeletal disorders, manual wheelchairs and crutches are commonly used for aiding locomotion. The wheelchair was fitted with an accessory board (90.0 cm × 15.0 cm × 1.0 cm) to prevent the sole surface of the fixed side from touching the floor (Figure 1). The non-fixed limb was placed on a foot support to limit movement during wheelchair locomotion; however, the movement of the non-fixed limb was not completely restricted. Toileting behavior was permitted for ethical considerations; however, patients were instructed to minimize the number of standing movements. The non-fixed side was fitted with thick-soled shoes to prevent the fixed side from touching the floor during standing movements. To evaluate numbness or pain, the participants’ legs were monitored hourly during cast application. The researcher promptly removed the bandage and splint once the immobilization period was over. However, the participants were not allowed to move their immobilized legs until the experiments were completed.

### 2.3. Examination of Standing Postural Stability

A single force plate (Twin Gravicorder G-6100; Anima Corp., Tokyo, Japan) was used to calculate the sway path of the COP during quiet standing. Force plate data were recorded at a sampling frequency of 20 Hz and were stored on a personal computer for subsequent analysis. A sampling frequency of 20 Hz is recommended by the Japanese Society of Equilibrium for clinical trials of gravitational COP sway, and Rhea et al. [19] reported no difference between the data obtained with a frequency of 20 Hz and that obtained with sampling frequencies of 100 Hz, 50 Hz, and 25 Hz.

Participants were instructed to stand barefoot at an approximately 30° angle and with their arms hanging relaxed at the side of their body without moving or speaking during the measurement. The experiment was conducted under open-eye (EO) or closed-eye (EC) conditions; the participants were instructed to look at a visual target placed 2 m in front of their eyes (visual angle, 1) during the EO condition. To eliminate the influence of outliers, the test was initiated 5 s after the participant stood on a single force plate. The recording time was 60 s and the recording started after the participant’s posture had stabilized. The COP was measured twice, once before cast application and again after cast removal, and the average value was calculated. To evaluate the postural stability, total anterior–posterior (A–P) and medial–lateral (M–L) path length (cm) and sway area (cm^2^) were calculated for each condition. The total path length (TPL) was calculated as the distance traveled along the two-dimensional axes in the A–P and M–L coordinates over 60 s. The A–P and M–L path lengths (PL_AP_ and PL_ML_) represent the motion on the AP and ML coordinate axes, respectively, observed over 60 s. The sway area was calculated as the inner area bounded by the outermost part of the TPL. To evaluate the directional specificity of the postural control, the mean COP displacements in the A–P (cm) and M–L (cm) directions (mean COP displacement_AP_ and mean COP displacement_ML_) were calculated. For the A–P axis, a negative (−) value indicates backward displacement, whereas a positive (+) value indicates forward displacement. For the M–L axis, negative (−) and positive (+) values indicate left-sided (immobilization side) and right-sided (non-immobilization side) displacement, respectively. Parameters derived from the COP trajectories obtained by force plate are considered the gold standards of balance evaluation [20].

### 2.4. Statistical Analysis

SPSS software (version 26.0; IBM Corp., Armonk, NY, USA) was used for all statistical analyses. The Shapiro–Wilk test was used to investigate the normal distribution of the dependent variable and to assess whether parametric testing was required. Since the test revealed a normal distribution of the dependent variable, Student’s *t*-test was used to compare the results after 10 h. For all analyses, the alpha value was a priori set at *p* < 0.05. Cohen’s d values (0.2, 0.5, and >0.8 indicate small, medium, and large effects, respectively) [21] were used to estimate effect sizes.

## 3. Results

**Figure 2:**
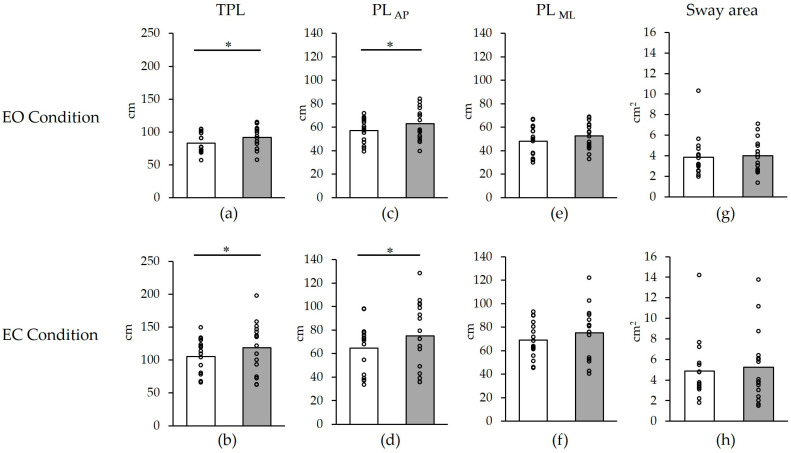
Changes in postural stability before and after cast removal. (**a**) TPL in EO condition; (**b**) TPL in EC condition; (**c**) PL_AP_ in EO condition; (**d**) PL_AP_ in EC condition; (**e**) PL_ML_ in EO condition; (**f**) PL_ML_ in EC condition; (**g**) Sway area in EO condition; (**h**) Sway area in EC condition. White: before; Gray: after 10 h. * *p* < 0.05. The plots show individual values for all participants. TPL, total path length; PL_AP_, anterior–posterior path length; PL_ML_, medial–lateral path length. EO, eyes open; EC, eyes closed. The graphs represent average values.

None of the participants dropped out of the study. Changes in postural stability before and after cast removal are shown in Figure 2 and Table 1. The TPL showed a significant increase from 83.2 ± 14.8 cm to 91.6 ± 16.5 cm in the EO test and from 105.4 ± 26.2 cm to 118.7 ± 37.5 cm in the EC test after cast removal compared to before the cast application (EO: *p* = 0.04, d = 0.54; EC: *p* = 0.04, d = 0.41) (Figure 2a,b). Similarly, the PL_AP_ significantly increased from 48.1 ± 12.3 cm to 52.6 ± 11.0 cm in the EO test and from 64.6 ± 20.6 cm to 75.2 ± 28.1 cm in the EC test after cast removal (EO: *p* = 0.01, d = 0.39; EC: *p* = 0.03, d = 0.43) (Figure 2c,d), whereas no significant changes were observed in the PL_ML_ (EO: *p* = 0.06, d = 0.50; EC: *p* = 0.10, d = 0.34) (Figure 2e,f). The sway area showed no significant change after cast removal (EO: *p* = 0.69, d = 0.10; EC: *p* = 0.51, d = 0.12) (Figure 2g,h).

**Figure 3:**
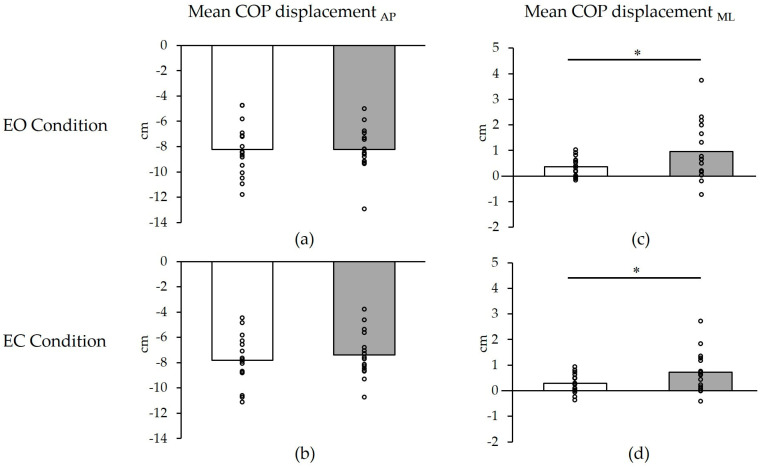
Changes in directional specificity of postural control before and after cast removal. (**a**) mean COP displacement_AP_ in EO condition; (**b**) mean COP displacement_AP_ in EC condition; (**c**) mean COP displacement_ML_ in EO condition; (**d**) mean COP displacement_ML_ in EC condition. White: before; Gray: after 10 h. * *p* < 0.05. The plots show individual values for all participants. The graphs represent average values. COP, center of pressure; AP, anterior–posterior; ML, medial–lateral; EO, eyes open; EC, eyes closed.

Changes in the directional specificity of postural control before and after cast removal are summarized in Figure 3 and Table 2. The mean COP displacement_ML_ significantly shifted from the immobilized (EO: 0.4 ± 0.4 cm; EC: 0.3 ± 0.4 cm) to the non-immobilized side (EO: 1.0 ± 1.1 cm; EC: 0.7 ± 0.8 cm) after cast removal (EO: *p* = 0.04, d = 0.72; EC: *p* = 0.03, d = 0.71) (Figure 3a,b), whereas the mean COP displacement_AP_ demonstrated no significant changes after cast removal (EO: *p* = 0.98, d = 0.01; EC: *p* = 0.30, d = 0.25) (Figure 3c,d).

## 4. Discussion

The present study investigated the acute effects of lower limb physical inactivity and movement restriction on postural sway in an upright posture after cast removal in healthy adults. The results showed that COP sway increased and that the COP position during the upright stance shifted from the immobilized to the non-immobilized side after cast removal compared to before the cast application. Since these significant differences were observed in healthy adults, changes in COP sway and directional specificity may be considered acute adverse events caused by movement restriction. Thus, the results affirm the findings of a previous study on the effects of postural sway during an upright stance after lower limb movement restriction [13].

The first main observation was that postural sway increased after cast removal compared to before the cast application. Previous short-term disuse studies have shown that movement restriction of the unilateral upper limb for 10 h in healthy adults reduces corticospinal tract excitability [22]. The corticospinal tract is considered responsible for proper muscle activity mobilization and postural coordination through the centrifugal transmission of information from the primary motor cortex [23]. If reduced corticospinal tract excitability occurs in the lower and upper limbs due to unilateral motor restriction, upright postural regulation may be compromised. Thus, the findings of the present study suggest that a stable posture can be compromised by changes in the excitability of the central nervous system rather than that of the peripheral nervous system. The second hypothesis concerns the influence of changes in somatosensory information processing. In humans, the sole is the only region in contact with the ground in an upright position. Thus, reduced postural stability can occur due to the inaccurate detection of somatosensory information from the sole [24]. Okamoto et al. [22] reported an increase in the amplitude of the N 30 component recorded in the frontal region during electrical stimulation of the median nerve after 10 h of motor restriction of a unilateral upper limb. Huber et al. [14] showed that after 12 h of movement restriction of the upper limb, the amplitude of the P 45 component recorded in the stimulated contralateral sensory motor cortex was reduced and the latency was increased compared to before the cast application. In another study, the amplitude of the brachial plexus-derived N 9 of short latency somatosensory evoked potentials increased 10 h after the cast immobilization of the unilateral upper limb [25]. The results of these studies are contradictory and debatable, establishing that changes in somatosensory evoked potentials can result in plastic changes in somatosensory information processing, even after only 10–12 h of immobilization of the upper limbs. Therefore, assuming that similar phenomena are induced in lower limbs, changes in somatosensory information processing may result in postural instability. These changes in somatosensory information processing may be due to reduced sensory input [26] and local circulatory disturbance [27], resulting from movement restriction using splints and medical bandages, and should be carefully monitored in future studies.

The second main observation was the shifting of the COP position during the upright stance from the fixed to the non-fixed side after cast removal. The COP contributes to the vertical projection of the center of mass and is considered to be the center point of all the pressure at the foot-ground contact surface [28]. The upright position of amputees [29], patients with hip osteoarthritis [30], and healthy adults with experimentally induced knee pain [31] has been shown to be biased toward the healthy side with a displaced M–L COP. Asymmetry in postural control can occur because of weight transfer from one leg to the other and plays an important role in maintaining body stability [32]. Therefore, the participants might have employed the most reliable non-fixed limb to control their standing posture and employed compensatory strategies to reduce postural sway. This effect may have increased the path length only in the anteroposterior direction.

The present study had some limitations. First, the relatively small sample size might have reduced the power of the results, rendering them less likely to be true [33]. Second, since the evaluations were conducted at different times on the same day, the results might have been affected by the confounding effect of diurnal variation [34,35]. Third, we did not record changes in brain activity, motor and sensory nerve activity, and regional circulation associated with changes in movement restriction; thus, whether these had an impact the COP sway remains unclear. Fourth, this study was conducted only on healthy adults; therefore, its clinical application is uncertain. Further studies are required to address these limitations and verify the results of the present study. 

## 5. Conclusions

The findings of the present study showed that postural sway increased and that the COP position during the upright stance shifted from the fixed to the non-fixed side after 10 h of wearing the cast compared to before the cast application. Changes in COP sway and directional specificity suggest acute adverse events caused by lower limb physical inactivity and movement restriction, confirming our hypothesis. Existing knowledge regarding the immediate effects of movement restriction suggests the need for acute rehabilitation for postural instability in patients following cast application. In future studies, the relationship between postural sway and weight-bearing asymmetry should be examined to clarify the basic mechanism of static postural control following exercise limitations.

## Figures and Tables

**Table 1:** Changes in postural stability before and after cast removal.

		Before	After 10 h	*p*-Values	d-Values
EO	TPL (cm)	83.2 ± 14.8	91.6 ± 16.5	0.04	0.54
	PL_AP_ (cm)	48.1 ± 12.3	52.6 ± 11.0	0.01	0.39
	PL_ML_ (cm)	57.2 ± 10.0	62.9 ± 13.1	0.06	0.50
	Sway area (cm^2^)	3.8 ± 2.0	4.0 ± 1.6	0.69	0.10
EC	TPL (cm)	105.4 ± 26.2	118.7 ± 37.5	0.04	0.41
	PL_AP_ (cm)	64.6 ± 20.6	75.2 ± 28.1	0.03	0.43
	PL_ML_ (cm)	68.9 ± 15.1	75.1 ± 21.6	0.10	0.34
	Sway area (cm^2^)	4.9 ± 2.9	5.2 ± 3.4	0.51	0.12

EO, eyes open; EC, eyes closed; TPL, total path length; PL_AP_, anterior-posterior path length; PL_ML_, medial–lateral path length. Average ± Standard deviations.

**Table 2:** Changes in directional specificity of postural control before and after cast removal.

		Before	After 10 h	*p*-Values	d-Values
EO	Mean COP displacement_AP_ (cm)	−8.2 ± 2.0	−8.2 ± 1.7	0.98	0.01
	Mean COP displacement_ML_ (cm)	0.4 ± 0.4	1.0 ± 1.1	0.04	0.72
EC	Mean COP displacement_AP_ (cm)	−7.8 ± 1.9	−7.4 ± 1.7	0.30	0.25
	Mean COP displacement_ML_ (cm)	0.3 ± 0.4	0.7 ± 0.8	0.03	0.71

EO, eyes open; EC, eyes closed; COP, center of pressure; AP, anterior–posterior; ML, medial–lateral. Average ± Standard deviations.

## Data Availability

The datasets used and analyzed in the current study are available from the corresponding author upon reasonable request.
